# Pulmonary hypertension subtypes associated with hereditary haemorrhagic telangiectasia: Haemodynamic profiles and survival probability

**DOI:** 10.1371/journal.pone.0184227

**Published:** 2017-10-05

**Authors:** Sabine Revuz, Evelyne Decullier, Isabelle Ginon, Nicolas Lamblin, Pierre-Yves Hatron, Pierre Kaminsky, Marie-France Carette, Pascal Lacombe, Anne-Claire Simon, Sophie Rivière, Jean-Robert Harlé, Alain Fraisse, Christian Lavigne, Vanessa Leguy-Seguin, Ari Chaouat, Chahera Khouatra, Sophie Dupuis-Girod, Eric Hachulla

**Affiliations:** 1 Département de Médecine Interne et Immunologie clinique, CHU de Brabois, Vandœuvre-lès-Nancy, France; 2 Pôle IMER, Hospices Civils de Lyon, Lyon, France; 3 Service d’Explorations Cardiologiques, Centre Hospitalier Lyon Sud, Pierre-Bénite, Hospices Civils de Lyon, Lyon, France; 4 Service de Cardiologie, CHRU de Lille, Université de Lille, Lille, France; 5 Service de Médecine interne, CHRU de Lille, Université de Lille, Lille, France; 6 Service de Radiologie, APHP Hôpital Tenon, Paris, France; 7 Service d’Imagerie diagnostique et interventionnelle, APHP Hôpital Ambroise Paré, Boulogne-Billancourt, France; 8 Service de Pneumologie, CHU Poitiers, Poitiers, France; 9 Service de Médecine interne, CHU Montpellier, Montpellier, France; 10 Service de Médecine interne, CHU Marseille Hôpital de la Conception, Marseille, France; 11 Paediatric Cardiology Service, Royal Brompton and Harefield Hospitals Trust, London, United Kingdom; 12 Service de Médecine interne et Maladies vasculaires, CHU Angers, Angers, France; 13 Service de Médecine interne et Immunologie clinique, CHU Dijon, Dijon, France; 14 Département de Pneumologie, CHU de Brabois, Vandœuvre-lès-Nancy, France; 15 Service de Pneumologie, Hôpital Louis Pradel, Hospices Civils de Lyon, Bron, France; 16 Service de Génétique et Centre de Référence pour la Maladie de Rendu-Osler, Hôpital Louis Pradel, Hospices Civils de Lyon, Bron, France; Odense University Hospital, DENMARK

## Abstract

**Background:**

Different pulmonary hypertension (PH) mechanisms are associated with hereditary haemorrhagic telangiectasia (HHT).

**Methods and results:**

We conducted a retrospective study of all suspected cases of PH (echocardiographically estimated systolic pulmonary artery pressure [sPAP] ≥ 40 mmHg) in patients with definite HHT recorded in the French National Reference Centre for HHT database. When right heart catheterization (RHC) was performed, PH cases were confirmed and classified among the PH groups according to the European guidelines. Among 2,598 patients in the database, 110 (4.2%) had suspected PH. Forty-seven of these 110 patients had RHC: 38/47 (81%) had a confirmed diagnosis of PH. The majority of these had isolated post-capillary PH (n = 20). We identified for the first time other haemodynamic profiles: pre-capillary pulmonary arterial hypertension (PAH) cases (n = 3) with slightly raised pulmonary vascular resistances (PVR), and combined post- and pre-capillary PH cases (n = 4). Compared to controls, survival probability was lower in patients with PAH.

**Conclusion:**

This study revealed the diversity of PH mechanisms in HHT. The description of combined post- and pre-capillary PH with/or without high cardiac output (CO) suggests either a continuum between the pre- and post-capillary haemodynamic profiles or a different course in response to high CO.

## Introduction

Hereditary haemorrhagic telangiectasia (HHT) is an autosomal dominant disease and displays age-related penetrance with increased manifestations developing over a lifetime. Diagnosis is based on the Curaçao criteria [1]. Prevalence in France ranges up to 1 in 8,500 [2]. Mutations in either the endoglin (*ENG*) or activin A receptor type II-like 1 (*ACVRL-1*) gene, encoding proteins that are components of the transforming growth factor-beta superfamily, account for most clinical cases [3,4]. Mutations in *MADH4* gene, encoding Smad 4, have also been described.

Rare cases of pulmonary hypertension (PH) in patients with HHT have been reported: post-capillary PH (Group 2), and, more rarely, pulmonary arterial hypertension (PAH) (Group 1) [5–14].

Group 2 PH is due to high cardiac output (CO) leading to left-heart failure, secondary to hepatic shunting, more frequent in patients with *ACVRL1* mutation [14]. *ACVRL1* mutation is reported to be a risk factor for the PAH cases (Group 1) [6,7,9].

The objectives of our study were: 1) to describe the different types of PH in an HHT population; 2) to identify predisposing factors by comparing the characteristics of each type of PH with those of a control population; 3) to compare survival probability in HHT patients according to the suspicion of PH and to its type when confirmed by right heart catheterization (RHC); and 4) to describe the phenotypic and genotypic characteristics of HHT patients with high estimated systolic pulmonary arterial pressure (sPAP) based on echocardiography (≥ 40 mmHg) and to compare them with those of a control HHT population with estimated sPAP < 40 mmHg.

## Methods

This retrospective, multicentre, descriptive study was based on data from the French national HHT database CIROCO (‘Clinical Investigation and Research for the Rendu-Osler Cohort’) in June 2014, provided by the French HHT reference centre (Lyon) and 13 French HHT skill centres. Data are recorded in the database either by each HHT specialist physician or by the National Reference Centre for HHT clinical research associate. Medical information is not available for all persons entered in the CIROCO database as relatives of patients can also be included. Possible or potential HHT cases can also be entered in this database: among the 5,628 cases reported in the French database as possible HHT cases or HHT relatives, 2,598 cases had a definite HHT diagnosis (i.e., fulfilled 3 or 4 Curaçao criteria or had an HHT pathogenic mutation). In accordance with French legislation, the approval of the *Commission nationale de l’informatique et des libertés* was obtained for this register. The approval of the ethics committee was not required. Patients’ anonymity was preserved.

### Patient selection

Among patients who received at least one echocardiography, those with PH reported in the CIROCO database at least once during follow-up were analyzed if they had definite HHT and an estimated sPAP, evaluated by echocardiography (regardless of the reason echocardiography was performed), ≥ 40 mmHg (based on peak tricuspid regurgitant jet velocity and right atrial pressure estimation) [15]. Patients with definite HHT diagnosis, reliable echocardiographic data (i.e., if sPAP ≥ 40 mmHg only once during follow-up without confirmation by other echocardiography or RHC, patients were not included) and information available on all the variables of interest were included in the study.

### Groups and PH types

**Fig 1:**
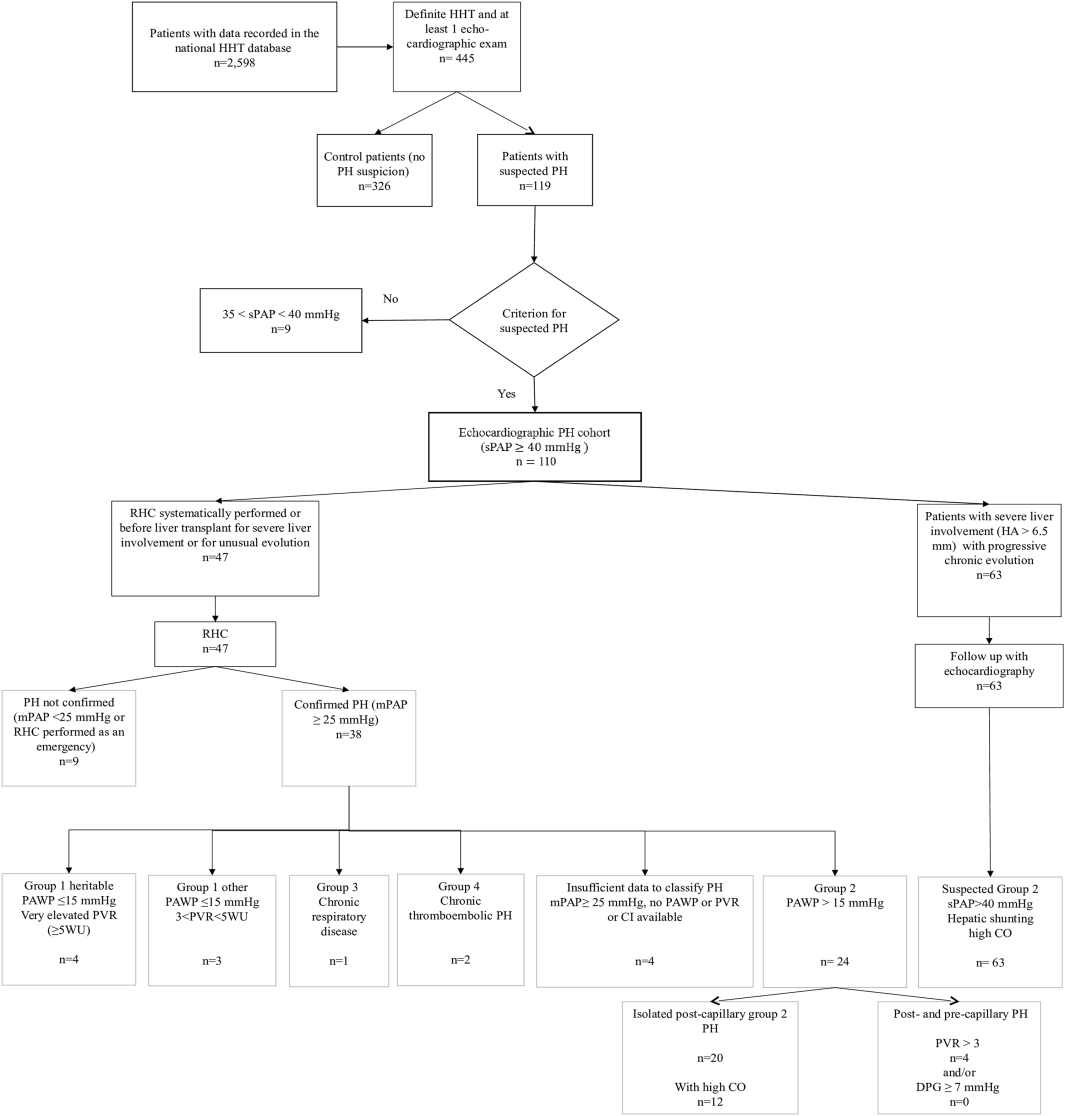
Flow chart of HHT patients selected for the study. CO, cardiac output; HA, hepatic artery; HHT, hereditary haemorrhagic telangiectasia; sPAP, systolic pulmonary artery pressure; mPAP, mean pulmonary artery pressure; dPAP, diastolic pulmonary artery pressure; PH, pulmonary hypertension; PVR, pulmonary vascular resistances; PAWP, pulmonary artery wedge pressure; RHC, right heart catheterization; WU, Wood units.

HHT diagnosis was based on the Curaçao criteria, namely at least 3 of the following signs: epistaxis, telangiectasia, first degree relative who meets diagnostic criteria, visceral signs [1]. Two nested case-control studies were done according to whether exploration was solely echocardiographic or also invasive haemodynamic based on the first data reported in the medical file (time between the first echocardiography and the RHC ranging from 0 to 998 days [median 32]):

- “Echocardiographic PH cohort”: echocardiographic suspicion of PH with or without confirmation by RHC (not systematically performed by some centres). The patients who underwent an RHC excluding the diagnosis of PH were included in this group.
- “RHC PH cohort”: PH confirmed by RHC. These patients were then classified into PH groups as defined in the latest European guidelines (Fig 1) [5].

When more than one echocardiography or RHC was performed, the first one available was considered if the first value seemed concordant with subsequent values, because there was no systematic echocardiography close to RHC.

PH was defined by a resting mean pulmonary arterial pressure (mPAP) ≥ 25 mmHg during RHC. Group 1 comprised patients with PAH (or heritable PAH, regardless of their *BMPR2* mutation status), defined by a pulmonary artery wedge pressure (PAWP) ≤ 15 mmHg and pulmonary vascular resistances (PVR) > 3 Wood units (WU). The PVR were calculated using the formula (mPAP – PAWP) / CO. Group 2 comprised patients with PH due to left heart disease, reflected by a PAWP > 15 mmHg. They were considered isolated post-capillary PH cases when the diastolic pressure gradient (DPG = dPAP – PAWP) was < 7 mmHg and PVR ≤3 WU. They were considered post- and pre-capillary PH cases when the DPG was ≥ 7 mmHg and/or PVR > 3 WU whatever the cardiac index, according to the latest European guidelines [5]. In parallel, we defined “presence of high CO” as a cardiac index at rest > 4.0 L/min/m^2^. Left ventricle end-diastolic pressure was not reported. Group 3 comprised patients with PH due to lung diseases. Group 4 comprised patients with chronic thromboembolic PH. All haemodynamic data were analysed retrospectively by three PH specialist physicians, regardless of the first interpretation.

“Liver vascular impairment” was defined as the presence of hepatic shunts, elevation of hepatic artery diameter or flow rate, lesions such as telangiectases, or nodular and focal hyperplasia, seen on echo-Doppler. “Significant hepatic vascular involvement” was defined as a hepatic artery diameter > 6.5 mm on at least one imaging examination [12,16]; “pulmonary vascular impairment” was defined as pulmonary arteriovenous malformations (AVMs) on computerized tomography scan; “gastrointestinal vascular impairment” was defined as gastrointestinal haemorrhagic telangiectases on endoscopy performed because of gastrointestinal haemorrhage or anaemia not explained by epistaxis; “neurological AVMs” were defined as AVMs in the central nervous system as seen on computed tomography scan or magnetic resonance imaging; and “anaemia” was defined as a haemoglobin level ≤ 12 g/dL in women and ≤ 13 g/dL in men. The number of telangiectases was estimated by each HHT specialist physician on specific body sites (ears, lips, tongue and hands).

A control population, among patients recorded in the database with no echocardiographic suspicion of PH, served for the evaluation of survival and risk factors.

### Data collection

Genotypic and phenotypic data were obtained from the CIROCO database and echocardiography and RHC data from the clinical files (S1 File).

### Statistical analysis

The 2 populations were matched by centre and date of diagnosis. As hepatic AVMs are known to be a major component of high CO leading to Group 2 PH and pulmonary AVMs are debated as an objection to the use of PAH-specific pharmacotherapies (for fear of raising an already high CO) and as severe PH makes pulmonary AVM embolization more hazardous, their presence was considered for the survival analysis [17,18]. Quantitative variables are presented as mean +/- standard deviation or as median and range and were compared using analysis of variance. Qualitative variables are presented as number and percentage and were compared using a Chi^2^ test (or Fisher’s exact test if the criteria for a Chi^2^ test were not fulfilled). Kaplan-Meier survival curves were done, any differences being tested using the Log-rank method. Multivariable analysis was not performed because there were too many missing data.

## Results

Despite current recommendations, only 445 patients had undergone an echocardiographic examination among 2,598 patients with an HHT diagnosis in the database as of June 2014. Echocardiography was performed as part of systematic screening, because of symptoms (dyspnoea, cardiac insufficiency) or because of pulmonary or hepatic AVMs. Of these 445 patients, 119 were suspected of having PH. One hundred and ten patients (4.2%) fulfilled our criteria for analysis (Fig 1), two of whom were children at the time of PH diagnosis. Among these 110 patients, 43 echocardiographic examinations were performed for systematic screening and 67 because of symptoms (54 for dyspnoea, 7 for fainting or chest pain and 29 for cardiac insufficiency). The control population comprised 326 patients.

### Echocardiographic and RHC data

Of the 110 patients suspected of PH, 47 (42.7%) underwent RHC (between 1991 and 2014, performed by at least 12 different cardiologist experts and using non-harmonized protocols). In 38/47 (81%) patients, PH was confirmed. Among the 47 patients who underwent RHC, only 11 had been exclusively screened for PH by echocardiography without any recorded symptoms and 9/11 had confirmed PH, all of them with Group 2 PH. Among the 63 patients followed-up exclusively by echocardiography, cardiac index as assessed by echocardiographic estimation was high for the 29/35 (82.9%) patients with available data.

### Characteristics

The characteristics of the 2 cohorts and controls are presented in Tables 1 and 2. The risk factors for PH occurrence not specific to HHT were identical in each population, as was the proportion of patients who underwent gene mutation screening.

**Table 1:** Comparison of phenotypic and genotypic characteristics between patients with echocardiographic suspicion of PH and controls.

Characteristics of cases (echocardiographic PH cohort with estimated sPAP ≥ 40 mmHg) and controls	Cases (n = 110) (%)	Controls (n = 326) (%)	p-value
Female	64 (58.2)	183 (56.1)	0.2382
Presence of epistaxis	98 (89)	319 (97.9)	0.3012
Presence of telangiectases	99 (90)	304 (93.3)	0.0968
No. of telangiectases median (min-max)	63 (3–695)	30 (1–869)	<0.0001
Pulmonary AVMs	34 (30.9)	135 (41.4)	0.0221
Liver vascular impairment	85 (77)	119 (36.5)	<0.0001
HA diameter >6.5 mm	65 (59)	84 (25.8)	<0.0001
Ascites present at least once	1 (0.9)	1 (0.3)	1.0000
Liver transplantation	7 (6.3)	2 (0.6)	0.0002
Neurological AVMs	8 (7.3)	24 (7.4)	0.8199
Gastrointestinal vascular impairment	39 (35.5)	53 (16.3)	0.0241
Tested for genetic mutation at least once	102 (92.7)	311 (95.4)	0.756
Mutation present (% of tested)	83 (81.4)	275 (88.4)	0.9470
Type of mutation (% of tested)			0.0001
*ACVRL1*	70 (68.6)	168 (54)	
*Endoglin*	12 (11.8)	102 (32.8)	
*MADH4*	1 (0.9)	5 (1.6)	
Current complications/medical history of thrombophlebitis	8 (7.3)	18 (5.5)	0.4925
Cardiac malformation	10 (9)	16 (4.9)	0.1030
Aortic malformation	1 (0.9)	5 (1.5)	1.0000
Autoimmune disease	1 (0.9)	4 (1.2)	1.0000

Figures are expressed as n (%) unless otherwise indicated. No., number; HA, hepatic artery; AVMs, arteriovenous malformations

**Table 2:** Comparison of phenotypic and genotypic characteristics between patients with confirmed diagnosis of PH and controls.

Characteristics of confirmed PH cases (RHC PH cohort with mPAP ≥ 25 mmHg) and controls	Cases (n = 38) (%)	Controls (n = 326) (%)	p-value
Female	23 (60.5)	183 (56.1)	0.6052
Presence of epistaxis	34 (89.5)	319 (97.9)	0.0196
Presence of telangiectases	35 (92.1)	304 (93.3)	1.0000
No. of telangiectases median (min-max)	40 (7–668)	30 (1–869)	0.1819
Pulmonary AVMs	15 (39.5)	135 (41.4)	0.3206
Liver vascular impairment	30 (78.9)	119 (36.5)	<0.0001
HA diameter >6.5 mm	21 (55.3)	84 (25.8)	0.0008
Ascites present at least once	1 (2.6)	1 (0.3)	0.3964
Liver transplantation	6 (15.8)	2 (0.6)	<0.0001
Neurological AVMs	2 (5.3)	24 (7.4)	1.0000
Gastrointestinal vascular impairment	14 (36.8)	53 (16.3)	0.4743
Tested for genetic mutation at least once	36 (94.7)	311 (95.4)	
Mutation present (% of tested)	31 (86.1)	275 (88.4)	0.4441
Type of mutation			0.0264
*ACVRL1*	26 (72.2)	168 (54)	
*Endoglin*	4 (11.1)	102 (32.8)	
*MADH4*	1 (2.8)	5 (1.6)	
Current complications/medical history of thrombophlebitis	2 (5.3)	18 (5.5)	1.0000
Cardiac malformation	5 (13.2)	16 (4.9)	0.0670
Aortic malformation	1 (2.8)	5 (1.5)	0.5073
Autoimmune disease	0	4 (1.2)	1.0000

Figures are expressed as n (%) unless otherwise indicated. RHC, right heart catheterization; No., number; HA, hepatic artery; AVMs, arteriovenous malformations

Whereas a greater number of telangiectases, a lower frequency of pulmonary vascular impairment and a higher frequency of gastrointestinal vascular impairment seemed to be associated with echocardiographic suspicion of PH, they were not significantly associated with the RHC PH cohort. Compared to controls, RHC PH cohort patients had fewer epistaxis episodes (p = 0.0196), more frequent and significant hepatic vascular involvement (p = 0.0008) and more frequently carried the *ACVRL1* mutation (p = 0.0264).

### Haemodynamic profiles

The haemodynamic characteristics of patients who underwent RHC are detailed in Tables 3 and 4. Within the RHC PH cohort, we identified 7/38 patients fulfilling the Group 1 PAH criteria. Among them, we distinguished 2 populations (with the exception of a young child [case No.106/f] whose haemodynamic profile and cardiac index are more difficult to understand). One case presented high CO and moderately elevated PVR (3 WU < PVR < 5 WU), a profile rarely reported in HHT. Four cases had very elevated PVR (≥ 5 WU), but only one with an altered cardiac index (<3.0 l/min/m^2^), as seen in heritable PAH. Twenty-four out of 38 patients fulfilled the criteria for Group 2, 1 patient for Group 3, 2 patients for Group 4 and 4 patients had insufficient data for them to be classified. Only 17/38 underwent ventilation/perfusion scanning, 7 of whom had a medical thromboembolic history.

**Table 3:** Haemodynamic profiles of patients in Group 1.

Case No./Sex	Age at PAH diagnosis	Mutation	sPAP	dPAP	mPAP	PAWP	RAP	PVR	CI	CO	Hb	HAVM	PAVM	follow-up (months)
42/f	67	*ENG*	66	22	39	9	8	4.55	3.95	6.59	12.8	yes	yes	10.5
57/f	35	*ENG*	79	32	48	13	12	3.57	4.4	9.8	10.4	yes	no	190.5
68/f	34	*ACVRL1*	76	21	47	5	6	5.7	4.1	7.35	13	yes	no	31.2
96/f	55	*SMAD4*	62	22	43	15	14	4.11	3.6	11.9	NK	yes	yes	9.9
105/f	29	*ACVRL1*	104	41	65	5	5	14.88	2.5	4.03	12	yes	yes	24
106/f	4	*ACVRL1*	83	48	65	9	11	10	5	5.6	14	no	yes	134.6
107/m	8	*ACVRL1*	58	29	40	10	8	6.4	NK	4.69	NK	no	no	119.7

CI, cardiac index; CO, cardiac output; dPAP, diastolic pulmonary artery pressure; *ENG*, *Endoglin*; f, female; HAVM, hepatic arteriovenous malformation; m, male; mPAP, mean pulmonary artery pressure; NK, not known; No., number; PAVM, pulmonary arteriovenous malformation; PAH, pulmonary arterial hypertension; PAWP, pulmonary artery wedge pressure; PVR, pulmonary vascular resistances; RAP, right atrial pressure; sPAP, systolic pulmonary artery pressure; WU, Wood units

**Table 4:** Haemodynamic profiles of patients in Group 2.

Case No./ Sex	Age at PH diagnosis	Mutation	sPAP	dPAP	mPAP	PAWP	RAP	PVR	CI	CO	Hb	HAVM	PAVM	follow-up (months)
5/f	53	*ENG*	55	28	35	28	15	0.93	4.28	7.5	NK	yes	yes	46.1
13*/f	78	*NK*	60	NK	40	NK	NK	NK	NK	NK	12	yes	no	11.3
14/f	57	*ACVRL1*	44	18	30	20	8	NK	NK	NK	NK	yes	no	90
21/f	64	*ACVRL1*	NK	NK	31	20	NK	NK	3.76	NK	12.9	yes	yes	109.6
22/m	66	*ACVRL1*	59	17	35	16	6	5.35	3.55	6.5	NK	yes	no	3.8
24/f	36	*ACVRL1*	50	NK	38	30	NK	NK	NK	NK	NK	yes	yes	226.2
28/m	64	*ACVRL1*	NK	NK	25	16	NK	NK	NK	NK	10	yes	no	94.6
29/f	41	*ACVRL1*	58	23	40	20	16	1.83	8.42	10.9	NK	yes	no	221.5
36/m	61	*ACVRL1*	88	33	53	28	21	3.2	4.35	NK	10.4	yes	no	42.9
44/f	65	*ACVRL1*	75	NK	45	20	NK	3.32	3.82	7.53	8.9	yes	no	59.8
45 †/f	58	*ACVRL1*	33	9	21	11	5	< 3	4.4		10.3	yes	no	99.1
50/f	66	*ACVRL1*	45	17	28	18	10	1.425	4.87	7.61	10.7	yes	no	77.7
55/m	24	*NK*	NK	NK	34	17	NK	NK	↑	NK	NK	yes	yes	169.7
56/m	62	*ACVRL1*	74	34	52	28	24	1.9	NK	12.4	11	yes	yes	15.7
58/m	65	*NK*	54	18	35	16	7	2.112	4.6	9	8.8	yes	no	14.7
59 ‡/m	64	*ACVRL1*	52	26	35	11	NK	2.29	NK	10.5	7	yes	no	130.1
60 §/m	63	*ACVRL1*	65	21	39	15	9	1.725	6.7	14	8.7	yes	no	26.7
65/f	54	*ACVRL1*	42	20	29	16	13	1.4	4.8	9.13	NK	yes	no	53.3
66/f	71	*ACVRL1*	65	23	44	24	17	3.6	3.5	5.57	12	yes	no	19.5
67/f	66	*ACVRL1*	73	25	44	23	18	2	6.4	10.43	7	yes	no	67
77| | /m	74	*NK*	49	18	30	15	5	1.6	5.2	9.5	9	yes	no	2.6
79/m	70	*ACVRL1*	NK	NK	32	16	18	1.65	5.71	NK	NK	yes	no	44.9
99/f	78	*NK*	51	19	32	17	NK	1.6375	4.6	6.72	8.6	yes	yes	60.1
109/m	64	*ACVRL1*	63	27	45	18	13	2	5.9	14.2	12	yes	no	10.2

CI, cardiac index; CO, cardiac output; dPAP, diastolic pulmonary artery pressure; *ENG*, *Endoglin*; f, female; HAVM, hepatic arteriovenous malformation; m, male; mPAP, mean pulmonary artery pressure; NK, not known; No. number; PH, pulmonary hypertension; PAVM, pulmonary arteriovenous malformation; PAWP, pulmonary artery wedge pressure; PVR, pulmonary vascular resistances; RAP, right atrial pressure; RHC, right heart catheterization; sPAP, systolic pulmonary artery pressure; WU, Wood units

^↑^ increased

These patients were considered group 2 PH despite the absence of loading test during RHC to distinguish PAH from PH:

* Considered Group 2 PH due to severe mitral stenosis.

^†^ RHC performed in 2005; first echocardiography 5 months before: PH suspected on sPAP 50 mmHg (45+5). Effort PH diagnosed (sPAP 53 mmHg /dPAP 19 mmHg /mPAP 35 mmHg after exercise) but the echocardiographic follow-up confirmed the group 2 PH suspicion. No further RHC was performed.

^‡^ RHC performed in emergency for cardiac insufficiency with high output failure before liver transplant. All echocardiographic parameters (including left atrial dilatation) were corrected after transplant, confirming Group 2 PH.

† and ‡: These two patients had elevated mPAP and normal PAWP, but PVR < 3 WU.

^§^ RHC performed before bevacizumab use. All echocardiographic parameters were corrected after bevacizumab injections.

^| |^ Considered Group 2 PH due to severe ischemic cardiomyopathy.

Within Group 2, 20/24 (83.4%) were isolated post-capillary PH. Cardiac index was high for 12 of the 13 (92%) patients with complete available haemodynamic data. The four other cases were post- and pre-capillary PH (defined as DPG ≥ 7 and/or PVR > 3 WU). One of them had a high cardiac index (mean 3.83 L/min/m^2^).

### PH estimated prevalence

**Fig 2:**
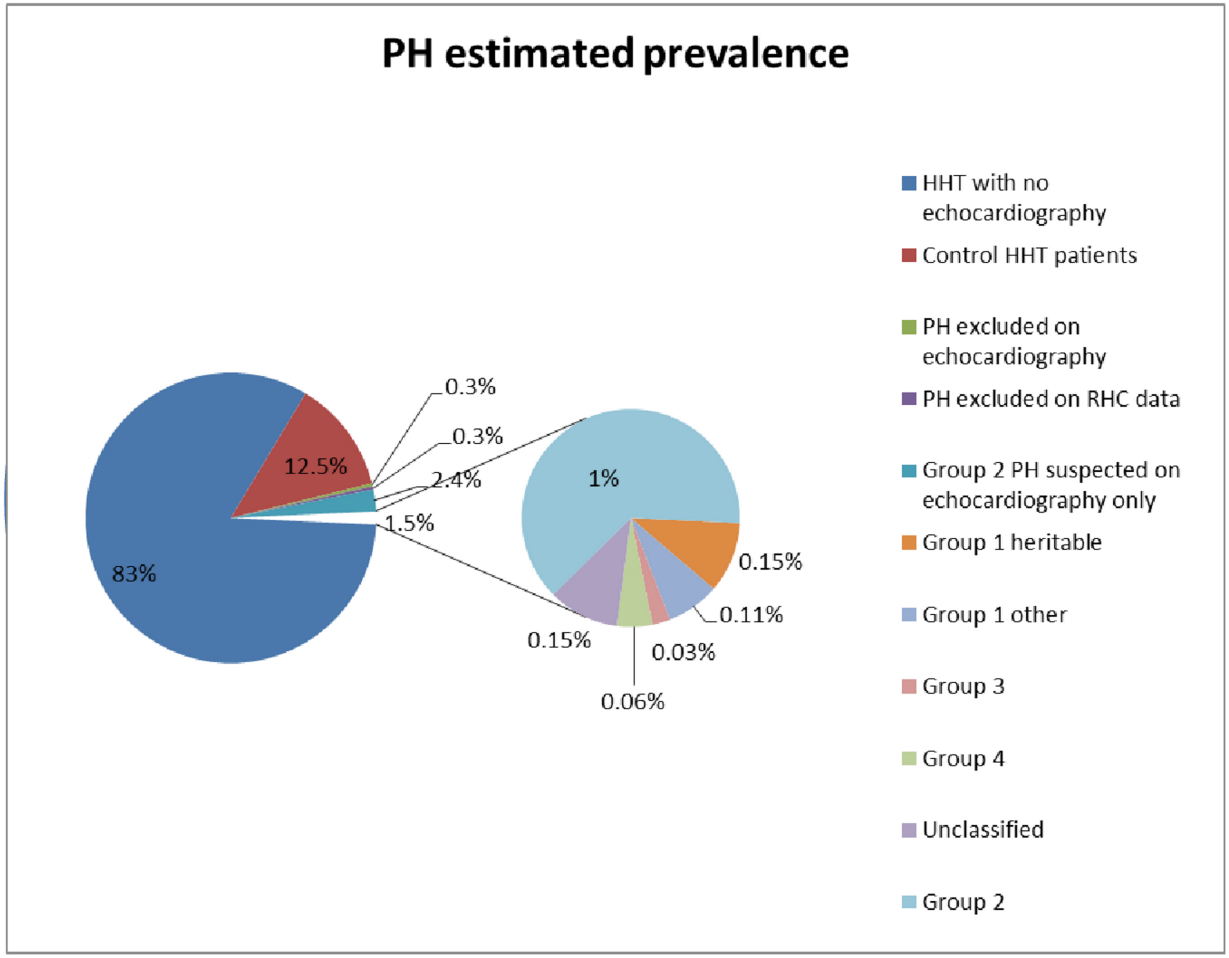
PH estimated prevalence. HHT, hereditary haemorrhagic telangiectasia; PH, pulmonary hypertension; RHC, right heart catheterization.

The overall occurrence of suspected PH was 4.23% (110/2,598 HHT patients); the minimum prevalence of confirmed PH (all groups considered together) was 1.5% (38/2,598), as shown in Fig 2. When RHC was performed, PH was confirmed in 38/47 (81%) cases.

### Treatment and survival comparisons

Five out of 7 patients in Group 1 were treated with PAH-specific pharmacotherapies and all patients in Group 2 were treated for high CO consequences (diuretics, salt restriction, atrial fibrillation management, etc.) and significant hepatic vascular involvement (using bevacizumab and/or in some cases liver transplant).

**Fig 3:**
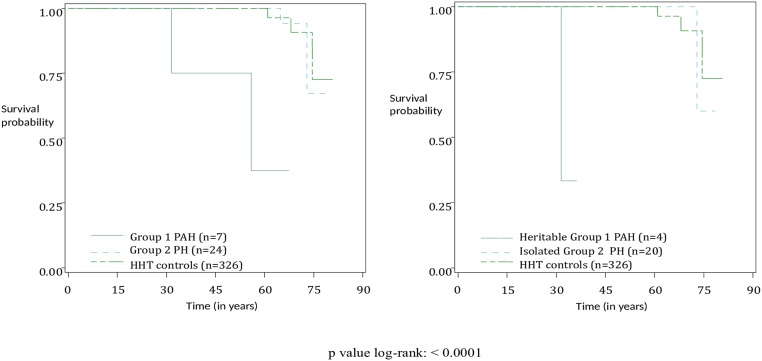
Comparison of life expectancy between Groups 1 and 2 and then between heritable Group 1 and isolated Group 2 patients with PH confirmed by RHC and controls with normal echocardiographic findings. HHT, hereditary haemorrhagic telangiectasia; PAH, pulmonary arterial hypertension; PH, pulmonary hypertension.

Patients with proven PAH seemed to have a lower life expectancy compared to controls (p = 0.0009). This seems to be due to heritable Group 1 PAH (p<0.0001) (Fig 3). Age at death and causes of death were collected in the database, as shown in Tables 5 and 6.

**Table 5:** Deaths among Group 1 patients: age at death and cause of death.

Case No.	Death	Age at death	Cause of death							
			nasal bleeding	cerebral bleeding	pulmonary embolism	cancer	PAH	sepsis	GI bleeding	other
42	no									
57	no									
68	no									
96	yes	55							yes	
105	yes	31					yes			
106	no									
107	no									

GI, gastro-intestinal; PAH, pulmonary arterial hypertension

**Table 6:** Deaths among Group 2 patients: age at death and cause of death.

Case No.	Death	Age at death	Cause of death							
			nasal bleeding	cerebral bleeding	pulmonary embolism	cancer	PH	sepsis	GI bleeding	other
5	yes	56						yes		
13	no									
14	yes	66								not known
21	yes	73					yes	yes		
22	no									
24	no									
28	no									
29	no									
36	yes	64						yes		
44	yes	72								car accident
45	no									
50	no									
55	no									
56	no									
58	no									
59	yes	75						yes		
60	no									
65	no									
66	no									
67	no									
77	no									
79	yes	73					yes			
99	no									
109	no									

GI, gastro-intestinal; PH, pulmonary hypertension

## Discussion

PH is a rare complication in the course of HHT; its prevalence is estimated to be < 1%, [7,8,19–21]. The frequencies and forms of PH have rarely been reported [22]. In this study, the overall occurrence of suspected PH in HHT patients was 4.23%. The minimum prevalence of confirmed PH (all groups considered together) was 1.5% [5]. Due to the lack of systematic echocardiographic examinations, this minimum prevalence is underestimated. When RHC was performed, PH was confirmed in 38/47 (81%) cases, which is comparable to data reported by Lyle et al. [22]. Group 2 PH (confirmed or highly suspected) accounted for 79% and heritable Group 1 for 3.6% of PH. We highlighted several mechanisms. As also described by Lyle et al. [22], we reported isolated Group 2 PH and heritable Group 1 PAH. Within group 2 PH, as also reported by Lyle et al. [22], 2 patients (cases 45 and 59) had elevated mPAP, normal PAWP and PVR < 3 WU, suggesting that PH can develop via high PAWP and/or high-flow state. These patients illustrate the difficulty in assigning patients to a specific group after only an RHC and the importance of follow-up. However, we distinguished 2 other groups. Most of the HHT patients had an isolated group 2 PH profile, but the description of more rarely described profiles (heritable Group 1 [4/38], Group 1 with high CO and moderately high PVR [1/38], post- and pre-capillary Group 2 [4/38]), may encourage clinicians to perform an RHC in patients with unclear echocardiographic patterns.

Despite the recommendations, only 445/2,598 HHT patients had undergone echocardiography, as recorded in the CIROCO database [23]. Data were not available on all variables of interest, including echocardiography, for every case reported in the database. Sixty-three patients out of 110 (57.2%) surprisingly never underwent RHC. These were patients with a history of progressive elevation of CO and left ventricle filling pressure, with high sPAP when decompensated. In such cases, sPAP is the marker of stable or unstable heart disease. The natural history of isolated Group 2 PH has been described elsewhere: high CO, leading to left heart impairment, the occurrence of PH, then right heart impairment [13,14]. Among 38 patients with confirmed PH, we described 24 (63%) Group 2 PH patients. Of these 24 patients, 17 had complete available haemodynamic data, 13 of whom (76.5%) had high CO. The threshold for cardiac index is debatable. Cardiac index measurement shows high variability data. Normal cardiac index is usually considered to be between 3.0 and 3.5 L/min/m^2^ but could be more during a stressful RHC [24]. In group 1 PAH, cardiac index is very rarely more than 3.5 L/min/m^2^ [25]. In group 2 PH, Ginon et al. defined high cardiac index as being superior to 3.8 L/min/m^2^ [14].

While echocardiography is useful for screening purposes, we consider invasive confirmation to be important [14]. In our series, in 9/47 (19%) patients, PH was not confirmed by RHC. The sPAP cut-off in echocardiography is a possible explanation, as is the variability of sPAP and its overestimation due to pulmonary AVMs, or the medical management (diuretics, salt restriction, transfusion or iron for anaemia) could mitigate left heart dysfunction and presence of PH. In three recent series of HHT patients, PH was suspected in respectively 13.5%, 17% and 45% of patients, but RHC values are scarce [26–28].

We identified 7 PAH (Group 1) cases, representing a minimum prevalence of 0.26%, higher than in the general population [8]. Patients with PAH and HHT are reported to be younger at diagnosis and to have a better haemodynamic profile (lower PVR, higher CO) than those with idiopathic or other heritable PAH, but a worse prognosis [9]. Our group included children, a finding that has already been reported [29]. We identified 2 subgroups: heritable PAH cases; and 2 cases, with slightly elevated PVR and high CO, more reminiscent of PAH related to congenital cardiopathies than of heritable PAH. Furthermore, we described 4 post- and pre-capillary PH cases. These different haemodynamic profiles are not explained by the variables that we studied. They might be explained by unproven porto-pulmonary hypertension [30], or could represent a subset of patients with *ACVRL-1* mutations that develop hepatic arteriovenous malformations, leading to high CO and group 2 PH, and have a simultaneous proliferative vasculopathy in the lung, leading to Group 1 PAH, or could be a consequence of the high CO. One might suppose that chronic exposure to increased flow could lead to a remodelling of the vascular bed, leading to an elevation of PVR and the occurrence of PAH.

Do these profiles correspond to different responses to the high CO? Prospective studies with haemodynamic monitoring will be needed to investigate this hypothesis.

We failed to find any new predisposing factor for PH occurrence [31]. We did not perform multivariable analysis because too many data were missing. Significant hepatic vascular involvement was correlated with a high CO failure, and therefore with Group 2 PH [13,14]. The higher prevalence of the *ACVRL1* mutation has been demonstrated both in its correlation with liver vascular impairment and in the rare cases of PAH [7,8,32]. The correlation between the number of telangiectases and PH might be linked to a similar mechanism of vascular remodelling or to the augmentation of CO but was not confirmed in patients with confirmed PH. Pulmonary vascular impairment was associated with suspected PH on echocardiography but not with proven PH on RHC. This might be due to the smaller sample size of proven PH. Pulmonary AVMs might also be a risk factor for overestimating sPAP on echocardiography and therefore be considered as not significant enough to perform an RHC.

Lastly, the worse survival would appear to be exclusively linked to that of heritable Group 1 PAH. In view of the different profiles of PH, their therapeutic management and prognosis, we consider it essential to propose an echocardiographic evaluation to patients with HHT with unexplained dyspnoea, signs of cardiac insufficiency, of PH or significant hepatic vascular involvement, and to confirm the diagnosis if the sPAP is ≥ 40 mmHg [33–38]. Furthermore, it has been reported that PAH-specific pharmacotherapies can be deleterious in HHT patients with Group 2 PH.

Our study has several limitations. The first is related to the retrospective design of the study, namely missing data in the register and operator-dependent techniques. Some haemodynamic data (left ventricle end-diastolic pressure or loading test during RHC) are missing; they would have helped to better assign patients in Group 1 or Group 2 PH. There could be an overlap of patients between the post- and pre-capillary Group 2 PH and the Group 1 with high CO and moderately high PVR. The absence of systematic ventilation/perfusion scanning means that we were unable to exclude cases of Group 4 PH. Although we excluded 2 patients from haemodynamic classification as RHC was performed in emergency for severe cardiac insufficiency, there could still be a bias for RHC performed in more severe or symptomatic cases. The second limitation is due to the lengthy period of study, during which changes in practices and testing may have occurred. The third limitation is due to the lack of harmonized practices (frequency of screening for complications, differences in RHC protocols). Furthermore, we were unable to evaluate the true prevalence of suspected PH in HHT. However, all patients had a clinical evaluation. Despite the screening recommendations for pulmonary AVMs, only 445 patients underwent echocardiographic screening, as shown in the database. Nonetheless, this is the largest cohort reported to date [39,40]. Another limitation, resulting in a major selection bias, is that only 43% of the patients with suspected PH underwent RHC. With the development and improvement of echocardiography, the need for RHC has decreased. This limits our results on the frequencies of the haemodynamic forms of PH. Performing RHC more systematically in HHT would help to understand the mechanisms of PH. A standardized protocol of evaluation should be proposed, as has been done for other pathologies presenting this risk [41–44].

In conclusion, group 2 PH is far more frequent than other groups, but several mechanisms can lead to PH. Before treating HHT patients with PH, it is important to look for significant hepatic vascular involvement and to elucidate the mechanisms. PH could be screened for by echocardiography in the case of unexplained dyspnoea and be confirmed by RHC, with the same protocol being used in each centre. For HHT patients, echocardiography could be performed once every 5 years and once a year in the case of hepatic AVMs. Prospective studies are required to explore the physiopathology of the occurrence of these new haemodynamic profiles, the respective proportions of each PH group and differences in prognosis.

## Supporting information

S1 File Echocardiography and RHC anonym data. (XLSX)
